# Adverse Effects of Oral Nonselective and cyclooxygenase-2-Selective NSAIDs on Hospitalization for Acute Kidney Injury: A Nested Case–Control Cohort Study

**DOI:** 10.1097/MD.0000000000002645

**Published:** 2016-03-07

**Authors:** Chia-I. Chou, Chia-Jen Shih, Yung-Tai Chen, Shuo-Ming Ou, Chih-Yu Yang, Shu-Chen Kuo, Dachen Chu

**Affiliations:** From the Department of Otolaryngology-Head and Neck Surgery, Mackay Memorial Hospital (C-IC); School of Medicine, National Yang-Ming University, Taipei (C-JS, Y-TC, S-MO, C-YY, S-CK); Deran Clinic, Yilan (C-JS); Division of Nephrology, Department of Medicine, Taipei City Hospital, Heping, Fuyou Branch (Y-TC); Division of Nephrology, Department of Medicine, Taipei Veterans General Hospital, Taipei (S-MO, C-YY); National Institute of Infectious Diseases and Vaccinology, National Health Research Institutes, Miaoli County (S-CK): Division of Infectious Diseases, Taipei Veterans General Hospital (SC-K); Institute of Public Health and Community Medicine Research Center, National Yang-Ming University (DC); Department of Health Care Management, National Taipei University of Nursing and Health Sciences (DC); and Department of Neurosurgery, Taipei City Hospital, Taipei, Taiwan (DC).

## Abstract

To investigate the association between the use of nonselective or cyclooxygenase (COX)-2-selective nonsteroidal antiinflammatory drugs (NSAIDs) and risk of acute kidney injury (AKI) in a general Asian population.

We conducted an observational, nationwide, nested case–control cohort study using Taiwan's National Health Insurance Research Database between 2010 and 2012. AKI cases were defined as hospitalization with a principle diagnosis of AKI. Each case was matched to 4 randomly selected controls based on age, sex, and the month and year of cohort entry. Odds ratios (ORs) were used to demonstrate the association between hospitalization for AKI and current, recent, or past use of an oral NSAID.

During the study period, we identified 6199 patients with AKI and 24,796 matched controls. Overall, current users (adjusted OR 2.73, 95% confidence interval [CI] 2.28–3.28) and recent users (adjusted OR 1.17, 95% CI 1.01–1.35) were associated with increased risk of hospitalization for AKI. The risk was also similar for nonselective NSAIDs. However, neither current nor recent use of COX-2 inhibitors was significantly associated with AKI events.

Our study supported that the initiation of nonselective NSAIDs rather than COX-2 inhibitors is associated with an increased risk of AKI requiring hospitalization. Future randomized trials are needed to elucidate these findings.

## Abstract 2

Supplemental Digital Content is available in the text

## INTRODUCTION

Nonsteroidal antiinflammatory drugs (NSAIDs), commonly used medications in the US,^1^ alleviate pain and inflammation associated with medical disorders by inhibiting isoenzymes of cyclooxygenase (COX): COX-1 and COX-2.

However, the adverse events, particularly gastrointestinal (GI) bleeding and renal dysfunction, are well-recognized in many nonselective NSAIDs because COX-1 inhibition impaired gastric mucosa integrity and renal hemodynamics. Thus, COX-2-selective NSAIDs theoretically were associated with less clinic GI and renal toxicity, whereas the benefits must be weighed against possible increased risks of cardiovascular events.^2,3^

The superior GI safety profile of COX-2-selective NSAIDs had been documented in previous studies,^4,5^ but the risk of acute kidney injury (AKI) among users of COX-2-selective NSAIDs remained controversial. Meta-analyses showed that the association of COX-2-selective NSAIDs with the risk of AKI did not achieve a statistical significance,^6^ or even existed only for Rofecoxib, but not for a COX-2 inhibitor class effect.^7^ Given AKI requiring hospitalization is relatively rare adverse renal events for NSAID users,^8^ population-based observational studies were encouraged to assess this infrequent adverse effect.

To date, we are aware of only few studies which have examined the AKI risk association of COX-2-selective NSAIDs, and most of the studies involve small samples or limited AKI events. We conducted a nationwide, nested case–control population-based study to evaluate the time-dependent association of NSAID use (nonselective or selective) with AKI and specially focus on differences in risk for various COX-2-selective NSAIDs, by using Taiwan's National Health Insurance Research Database (NHIRD).

## METHODS

### Data Sources

Taiwan's NHIRD is a prospectively recorded claims database, which contained information on all hospital admissions, out-patient visits, diagnoses, prescriptions, and procedures of 99.9% of 23 million inhabitants in Taiwan. The details of NHRD have been described previously.^9,10^ All diagnoses are recorded according to International Classification of Disease, ninth revision, Clinical Modification (ICD-9-CM). We used the Longitudinal Health Insurance Database dataset containing complete data of 1,000,000 randomly sampled beneficiaries during 1996 to 2010 from the original NHIRD. The dataset used in this study consists of deidentified secondary data exclusively for research purposes. As the patient information is encrypted in NHIRD, this study was exempted from a full ethical review by the institutional review board of Taipei City Hospital.

### Settings and Participants

This Taiwanese population aged ≥20 years, who were followed from 1 January 2000 to 31 December 2010, consisted of cases diagnosed with AKI and matched controls. Cases were defined as patients who were hospitalized with a principle diagnosis of AKI (ICD-9-CM 584.9), and the date of hospitalization was defined as the index date. Patients with history of chronic kidney disease (ICD-9-CM 250.4, 403, 404, 405.01, 405.11, 405.91, and 580–588) and kidney transplantation recipients were excluded. A pool of potential eligible controls with the same follow-up period as the case without a previous ICD-9 code for AKI was extracted from the Longitudinal Health Insurance Database. From these eligible controls, 4 were selected randomly and matched to a case by age (±1 year), sex, and the month and year of cohort entry. Charlson comorbidity index score,^11^ predisposing factors, or associated comorbidities for AKI including hypertension, diabetes mellitus, chronic liver disease, heart failure, coronary artery disease, dyslipidemia, autoimmune disease, drug abuse, peripheral vascular disease, cerebrovascular disease, gout, nephrolithiasis, and cancer (database codes shown in Supplementary Table 1), and concomitant drugs including angiotensin-converting-enzyme inhibitor, angiotensin II receptor blocker, beta-blocker, statin, steroid, and other nephrotoxic agents^12^ were also included in our analysis.

### Exposure Assessment

We identified all oral NSAIDs (including nonselective and COX-2-selecitve) prescribed in the year before the index date. The available COX-2-selecitve NSAIDs in Taiwan during the study period included celecoxib, etoricoxib, and rofecoxib. NSAID uses based on the timing between the prescription termination date (date of dispensation plus number of days of supply) and the index date were allocated to current, recent, or past use.^13,14^ We defined current users as those diagnosed with AKI during the NSAID prescription period, recent users as those diagnosed with AKI 1 to 30 days after the prescription termination date, and past users as those diagnosed with AKI at 31 to 180 days after the prescription termination date. We characterized the association of NSAID with AKI according to their COX-2 selectivity and different types of COX-2 inhibitors.

### Statistical Analysis

The demographic characteristics of the cases and controls were compared with Chi-square tests for categorical variables, and with the independent *t*-test for continuous variables. Odds ratios (ORs) were used to compare the exposure to NSAIDs among those with AKI and controls. Conditional logistic regression was used to adjust for confounding. In addition, given the varied degrees of safety concern of different NSAIDs on heart failure that may cause selection bias,^2^ we also conducted subgroup analyses that only enrolled subjects without heart failure. The Microsoft SQL Server 2012 (Microsoft Corp., Redmond, WA) was used for data linkage, processing, and sampling. All analyses were performed using STATA statistical software (version 12.0; StataCorp, College Station, TX) with 2-sided tests of significance at the *P* value level of <0.05.

## RESULTS

During the study period, 6199 patients with AKI and 24,796 matched controls were identified. Table 1 depicts the demographic characteristics and comorbidities of the cases and controls. The mean age was 67.1 years and predominately male (62.1%). Patients with AKI were more likely than controls to have hypertension, diabetes mellitus, chronic liver disease, and heart failure.

**TABLE 1:**
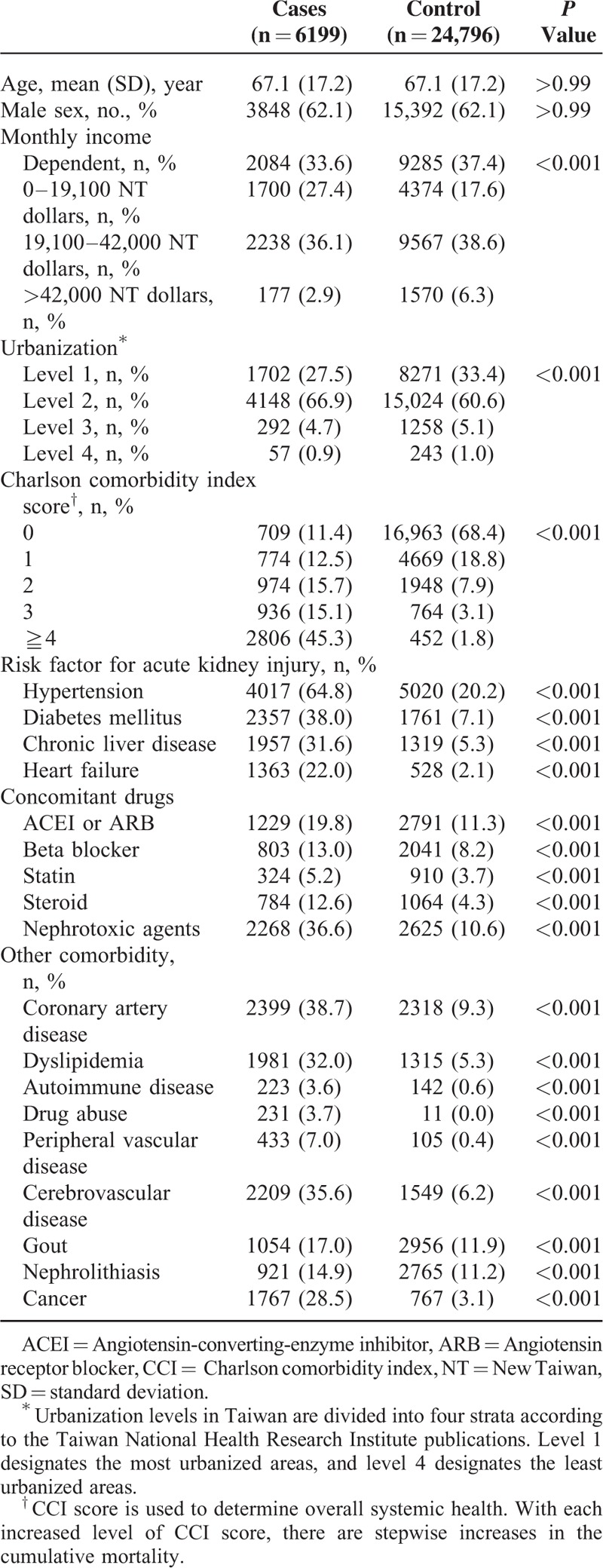
Characteristics of the Cases and Controls

Table 2 presents the crude and adjusted ORs (aORs) for the NSAID use (including nonselective and COX-2-selecitve NSAIDs) in cases who had diagnosis of AKI compared to controls by adjusting for all observed variables. In the nested case–control analysis, the risk of hospitalization for AKI was higher among current users (adjusted OR 2.73, 95% confidence interval [CI] 2.28–3.28) than recent users (adjusted OR 1.17, 95% CI 1.01–1.35), whereas past users (adjusted OR 0.62, 95% CI 0.55–0.70) were not associated with increased risk of AKI. In further analysis restricted to nonselective NSAIDs users or COX-2-selective NSAIDs (Table 3), similar association between nonselective NSAIDs and risk of AKI was observed, but adjusted ORs were consistently closer to null in current (adjusted OR 0.98, 95% CI 0.66–1.46) and recent (adjusted OR 1.07, 95% CI 0.65–1.76), although significant in past (adjusted OR 0.74, 95% CI 0.57–0.94) users of COX-2-selective NSAIDs.

**TABLE 2:**
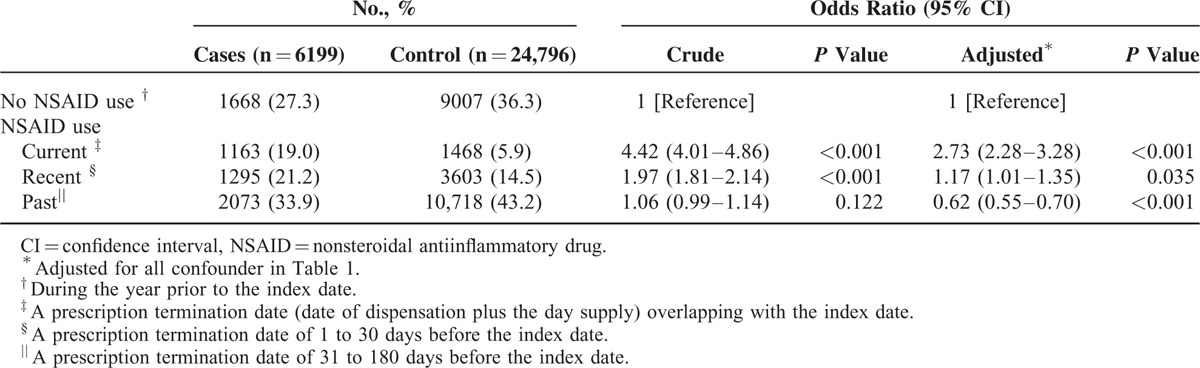
Crude and Adjusted Odds Ratios for the Risk of Hospitalization for Acute Kidney Injury With Oral NSAIDs

**TABLE 3:**
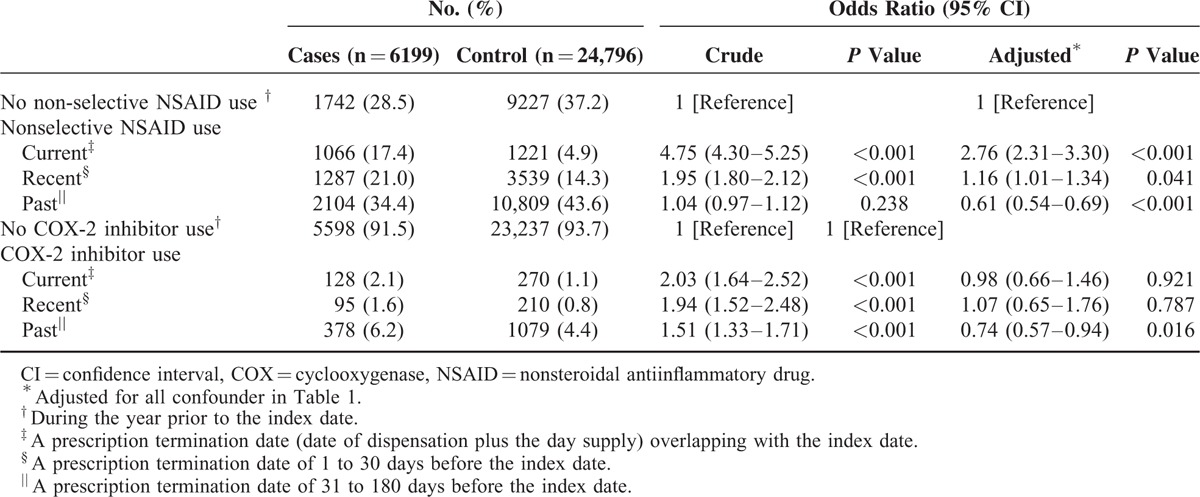
Crude and Adjusted Odds Ratios for the Risk of Hospitalization for Acute Kidney Injury With Different Type of NSAIDs

When stratified according to current use of individual NSAIDs with different perceived COX-2 selectivity (Table 4), the risk of AKI remained significantly increased in the subgroup analysis of patients taking nonselective NSAIDs with high COX-2 inhibition (adjusted OR 3.82, 95% CI 2.48–3.96) or other nonselective NSAIDs (adjusted OR 3.67, 95% CI 2.95–4.57), but the risk of AKI was insignificant for celecoxib (adjusted OR 1.07, 95% CI 0.67–1.72), etoricoxib (adjusted OR 0.39, 95% CI 0.15–1.05), or other relatively selective COX-2 inhibitor users (adjusted OR 0.79, 95% CI 0.51–1.22) although rofecoxib use was associated with increased risk of AKI (adjusted OR 3.32, 95% CI 1.23–9.01). In addition, subgroup analyses of nonselective or COX-2-selective NSAIDs users without heart failure also showed findings similar to the main analyses (Supplementary Table 2).

**TABLE 4:**
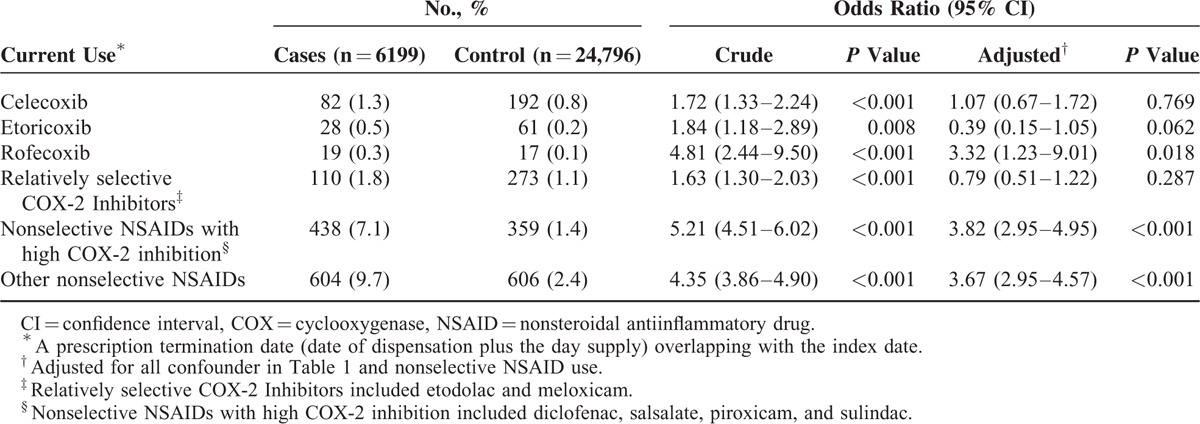
Crude and Adjusted Odds Ratios for the Risk of Hospitalization for Acute Kidney Injury With Current Use of Individual NSAIDs According to COX-2 Selectivity

## DISCUSSION

In this contemporary national representative NSAID nested-control cohort study, we found that the use of nonselective NSAIDs rather than COX-2-selective NSAIDs was associated with increased risk of hospitalization for AKI within 1 month of 1st prescription, although the risk was not increased in past users than unexposed controls, possibly due to confounding in baseline covariates between remote exposure and outcomes or representing a selected population less vulnerable to nephrotoxicity so that no more AKI events could be detected. However, the association of COX-2-selective NSAIDs subtypes with AKI was consistently insignificant for current use of celecoxib and etoricoxib, except for rofecoxib.

Our findings are concordant with those of previous meta-analysis^7^ of randomized controlled trials of COX-2 inhibitors and showed that only rofecoxib was associated with elevated risk of AKI, but no such association was observed for other COX-2 inhibitors. However, their findings needed to interpret with caution because of limited cases in each trial and potential publication bias. By contrast, a Canadian-based registry study demonstrated that elevated risk of AKI was evident among COX-2 inhibitor users (including rofecoxib and celecoxib) although the risk seemed to be insignificant for low-dose users.^12^ However, their results originated from older patients (mean age,78 years) than those in the present study (mean age, 67 years). Besides, a recent meta-analysis^6^ of large observational studies reported insignificant risk of AKI among different COX-2 inhibitor users, whereas in which most trials took place in more remote time periods, had small number of cases, and lacked the data from Asian ethnicity. Thus, in addition to recent emphasis of NSAID related cardiovascular harm by U.S. Food and Drug Administration in 2015 followed by the existing label warning in 2005,^15^ our study used a nationwide, unselected Taiwanese population to further strengthen the adverse effects of NSAID on AKI, which were mostly observed in nonselective NSAID users not in COX-2 inhibitor users and extended results of previous studies on different races.

The mechanisms responsible for the differential risk of AKI between nonselective NSAIDs or COX-2 inhibitors were beyond the scope of our design. Renal prostaglandin mainly synthesized by COX-1 served as an compensatory mechanism of renal hemodynamics.^16^ Thus, nonselective NSAIDs were theoretically more likely than COX-2 inhibitors to downregulate renal prostaglandin formation via inhibition of COX-1, leading to renal circulatory compromise. Previous some small studies also supported the evidences that COX-2 inhibitors spared more renal hemodynamic function than nonselective NSAIDs did,^17–19^ although some studies did not.^20,21^ Nevertheless, our results from 6199 AKI cases did provide a more robust basis for clarifying the controversy about whether COX-2 inhibitors were responsible for AKI events on a national scale.

The particular strengths of the present study include the use of prospectively recorded claims data reported by physicians, comprehensive follow-up (including outpatient visits and hospitalization), adjustment for important confounding factors, and use of reliable measures of NSAID exposure and AKI events. Nevertheless, our study has some limitations. The major one was confounding by indication from the retrospective observational pharmacoepidemiologic study design although we have adjusted for several covariates that may influence the AKI risk estimate. Thus, the current study only showed the association between NSAIDs and AKI, but no causal relationship can be either established or excluded. However, this type of cohort study is less time- and cost-consuming than a prospective trial because of relatively low incidence of AKI among NSAID users.^8^ Second, given that the information of serum creatinine changes was not available in our claims database, we used the strict definition for AKI based on validated ICD-9-CM coding of hospitalization diagnosis.^22^ Thus, the underestimated risk for AKI with mild severity (ie, those with AKI not seeking medical attention or requiring hospitalization) may be possible. Third, the use of over-the-count NSAIDs was not recorded in Taiwan's NHIRD. However, this bias may be negligible due to convenient medical access and minimal financial barrier of health insurance access in Taiwan. We also believed that the bias between 2 cohorts was nondifferential due to large sample size. Finally, general limitation of retrospective registry study included unavailable information on several potential confounding factors, including vital signs, body mass index, cigarette/alcohol consumption, nutritional condition, functional status, laboratory parameters, and family history of kidney disease.

## CONCLUSIONS

In a large “real-world” Asian population, the use of nonselective NSAIDs rather than COX-2 inhibitors is associated with an increased risk of AKI requiring hospitalization, especially within 1 month after prescription. Even though the present study was not a prospective, randomized study, present analysis resulted in a possibly less harmful effect of selective NSAIDs on kidney function. Future randomized trials are needed to elucidate these findings.

## Supplementary Material

Supplemental Digital Content
