# Supplementary material for: Adverse Effects of Oral Nonselective and cyclooxygenase-2-Selective NSAIDs on Hospitalization for Acute Kidney Injury: A Nested Case–Control Cohort Study
Source: Medicine (Baltimore). 2016 Mar 7;95(9):e2645. doi: 10.1097/MD.0000000000002645 (PMC4782836; doi:10.1097/MD.0000000000002645)
Supplement: Supplemental Digital Content [file medi-95-e2645-s001.pdf]

Supplementary Table 1. Characteristics and Database Codes

Characteristics	Database ICD-9 code
Hypertension	401.x – 405.x
Diabetes mellitus	250.x
Chronic liver disease	571.2,571.4,571.5,571.6,572.2-572.8
Heart failure	428.x
Coronary artery disease	411.x, 413.x, 414.x
Dyslipidemia	272.0, 272.1, 272.2, 272.4
Autoimmune disease	370.33, 710.x, 714.0, 720, 720.0
Drug abuse	304.x – 305.x
Peripheral vascular disease	443.9,441.x,785.4,V43.4
Cerebrovascular disease	430.x – 438.x
Gout	274.9
Nephrolithiasis	592.x
Cancer	140.x – 208.x

Supplementary Table 2. Crude and Adjusted Odds Ratios for the Risk of Hospitalization for Acute Kidney Injury With Different Type of NSAIDs in Patients Without Heart Failure

	Odds Ratio (95% CI)			
	Crude	P Value	Adjusted*	P Value
**No non-selective NSAID**	1 [Reference]		1 [Reference]	

**use** [†]				
**Non-selective NSAID use**				
**Current** [§]	5.06 (4.52-5.66)	<0.001	2.94 (2.42-3.57)	<0.001
**Recent** [ǁ]	1.98 (1.80-2.17)	<0.001	1.25 (1.07-1.47)	0.005
**Past** [¶]	1.00 (0.93-1.09)	0.925	0.61 (0.53-0.69)	<0.001
**No COX-2 inhibitor use** [†]	1 [Reference]		1 [Reference]	
**COX-2 inhibitor use**				
**Current** [§]	2.14 (1.66-2.74)	<0.001	0.94 (0.60-1.47)	0.794
**Recent** [ǁ]	1.83 (1.36-2.47)	<0.001	0.93 (0.52-1.68)	0.820
**Past** [¶]	1.44 (1.24-1.69)	<0.001	0.71 (0.53-0.95)	0.023

Abbreviations: NSAID, non-steroidal anti-inflammatory drugs; CI, confidence interval; COX, cyclooxygenase.

[*] Adjusted for all confounder in Table 1.

[†] During the year prior to the index date.

[‡] A prescription termination date (date of dispensation plus the day supply) overlapping with the index date.

[§] A prescription termination date of 1 to 30 days before the index date.

[ǁ] A prescription termination date of 31 to 180 days before the index date.
